# Preliminary investigations of parasite contamination of water sources in Armenia

**DOI:** 10.1016/j.fawpar.2024.e00221

**Published:** 2024-01-21

**Authors:** Oleg V. Shcherbakov, Sargis A. Aghayan, Hasmik Sh. Gevorgyan, Tigran A. Abgaryan, Ruzanna H. Gevorgyan, Alejandro Jiménez-Meléndez, Lucy J. Robertson

**Affiliations:** a Scientific Center of Zoology and Hydroecology, NAS RA. 7 P. Sevak str., 0014 Yerevan, Armenia; b Armenian National Agrarian University, 74 Teryan str., 0009 Yerevan, Armenia; c Parasitology, Norwegian University of Life Sciences (NMBU), Faculty of Veterinary Medicine, P.O. Box 5003, NO-1432 Ås, Norway

**Keywords:** Armenia, *Cryptosporidium*, *Giardia*, Sediment, Water contamination

## Abstract

The intestinal protozoan parasites, *Cryptosporidium* and *Giardia*, are known to have a global distribution, infecting and causing disease in a range of hosts, including people, livestock, pets, and wildlife. However, data from some regions is very sparse. In Armenia, in the Caucasus region of West Asia, only scanty data are available, with just a few surveys on *Cryptosporidium* infections in livestock, and no available data on human infections or environmental contamination. As part of implementation of water analysis methods for these parasites in Armenia, 24 raw water samples and two sediment samples were analysed for these parasites using a range of approaches, including modified Ziehl-Neelsen, Lugol stain, immunofluorescent antibody test (IFAT), qPCR and, on sediment samples, immunomagnetic separation and IFAT. Results suggest substantial contamination of raw water sources and indicate the need for further targeted studies using appropriate methods and collecting data on host infections in catchment areas.

## Highlights

• Information on *Cryptosporidium* and *Giardia* in Armenia is sparse.
• We investigated raw water and sediment samples collected from across Armenia.
• Detection techniques included conventional staining, IFAT, and qPCR.
• High detection suggests the need for investigation of both parasites in Armenia.

## 1 Introduction

The intestinal protozoan parasites *Cryptosporidium* and *Giardia* are transmitted when susceptible individuals ingest their robust transmission stages, oocysts and cysts, respectively, that are shed in the faeces of infected people and animals. Although transmission to people is probably mostly direct (hand-to-mouth), ingestion via contaminated water also occurs commonly. This may result in community-wide outbreaks should a municipal drinking water supply be contaminated with these parasites, resulting in a substantial disease burden (Gerdes et al., 2023).

In Armenia, a landlocked country in the Caucasus region of West Asia, there is very little information available regarding these parasites. Cryptosporidiosis is not a reportable infection in Armenia, and data from diagnostic laboratories is unavailable. The last investigation of human infection with *Cryptosporidium* was conducted over 10 years ago in Vanadzor city, Armenia's 3rd largest city, using both modified Ziehl-Neelsen (mZN) and commercial ELISA (RIDASCREEN® Cryptosporidium), and indicated a prevalence of around 7% (by mZN) and 17% by ELISA in patients with gastrointestinal complaints; peak infection occurred in September (Davidyants, 2011). Similar data on human *Giardia* infection are not available.

Among animals, two surveys have been conducted in farm animals, one from around 20 years ago and one from over 10 years ago, both using mZN. The first reported detection of *Cryptosporidium* shedding in around 23% of sheep samples, 31% of cattle samples, and 50% of pig samples from three regions of Armenia, with positive samples from younger animals (Naghashyan and Shcherbakov, 2006). The second study (Naghashyan et al., 2012), reported similarly high occurrences in four regions of Armenia and also suburbs of Yerevan, with around 22% of lambs, 25% of calves, and 20% of piglets positive for *Cryptosporidium*, but no detection in rabbits and chickens. Highest rates of detection were during the autumn. Data on *Giardia* infection in animals are not available.

Despite the apparently relatively high rates of *Cryptosporidium* infection in people and animals in Armenia, investigations on risk factors for infection are lacking, and there has been minimal investigation of the potential for waterborne transmission. An analysis of drinking water in Yerevan during the mid-1990s (American University of Armenia, 1996) found faecal coliform levels indicative of sewage contamination in some samples, but *Giardia* cysts were not detected; the analysis method for *Giardia* was not provided.

The purpose of our study was to provide preliminary data on contamination of raw water in different areas of Armenia with *Cryptosporidium* and *Giardia* during implementation of water analysis methods.

## 2 Materials and methods

### 2.1 Sample sites and site information

**Fig. 1:**
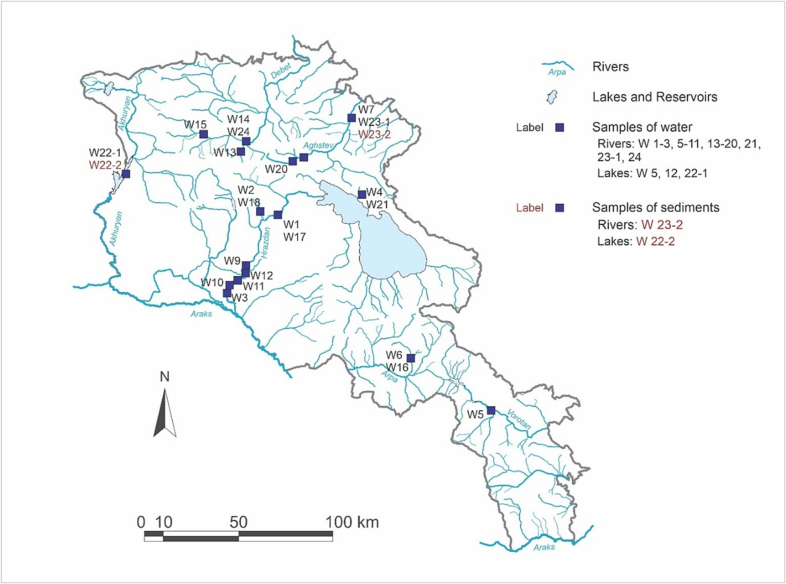
Map of the sampling points in Armenia showing sampling sites by sample codes and matrix type (water or sediment). Blue lines indicate the major rivers and streams. Squares with black labels indicate water samples; squares with brown labels indicate sediment samples (for numbering of the samples see Table 1). (For interpretation of the references to colour in this figure legend, the reader is referred to the web version of this article.)

We collected 24 samples of water of 10 L volume from 16 sites. The sites were chosen on the basis of being either agricultural areas, watered by large rivers that flow through towns/cities (*n* = 7) or recreational areas where people swim (*n* = 9); two sites were both recreational and agricultural. The sampling sites are located throughout Armenia, covering nine regions, including Yerevan, of Armenia's 11 regions (Fig. 1). Water types sampled were river water (13 sampling sites: one river with five sampling sites, two rivers with two sampling sites, and four rivers with one sampling site) and lake water (sample sites at one natural lake and two artificial lakes). None of the sites are directly used for production of drinking water.

Water samples were collected from July 2021 until November 2022, with no collection during the winter months (November until end of March). In addition, samples of sediment were collected (one river and one lake) in October and November 2022. See Table 1 for further details.

**Table 1:** Details of sampling sites for both water samples and sediment samples.

Sample Code	Sample type & water body	Location	Catchment use	Sampling Date	Altitude, MASL^b^	Coordinates
W-1	Water, River	**River Hrazdan**: Kotayk Marz, Bjni	Recreational, Agricultural	21.07.2021	1440	40°26′13″N; 44°37′14″E
W-2	Water, River	**River Dalar**: Kotayk Marz, Aghveran	Recreational	21.07.2021	1600	40°29′05″N; 44°35′47″E
W-3	Water, River	**River Hrazdan**: Ararat Marz, Darbnik; site is downstream of Yerevan STW^a^	Agricultural	22.07.2021	860	40°06′18″N; 44°22′49″E
W-4	Water, Lake	**Lake Sevan:** Gegharkunik Marz, Shoghakat	Recreational	14.08.2021	1903	40°29′09″N; 45°16′47″E
W-5	Water, River	**River Vorotan:** Syunik Marz, Sisian	Agricultural, Recreational	09.09.2021	1622	39°32′28″N; 46°00′43″E
W-6	Water, River	**River Herher**: Vayots Dzor Marz, Herher;	Recreational	09.09.2021	1333	39°41′21″N; 45°31′18″E
W-7	Water, River	**River Aghstev**: Tavush Marz, Ijevan; site is downstream of Ijevan & Dilijan towns	Recreational	08.10.2021	632	40°55′32″N; 45°09′17″E
W-8	Water, River	**River Aghstev**: Tavush Marz, Dilijan; site is upstream of Dilijan and Ijevan towns	Recreational	08.10.2021	1331	40°44′25″N; 44°49′41″E
W-9	Water, River	**River Hrazdan**: Children's Railway; site is upstream of Yerevan STW	Recreational	30.03.2022	960	40°11′12″N; 44°30′01″E
W-10	Water, River	**River Hrazdan**: Ararat Marz, Khachpar; site is downstream of Yerevan STW	Agricultural	30.03.2022	879	40°07′14″N; 44°24′22″E
W-11	Water, River	**River Hrazdan**: Ararat Marz, Argavand; site is upstream of Yerevan STW	Agricultural	30.03.2022	910	40°09′18″N; 44°26′42″E
W-12	Water, Lake	**Yerevan Lake**: site is upstream of Yerevan STW	Recreational	30.03.2022	933	40°09′42″N; 44°28′28″E
W-13	Water, River	**River Vanadzor**: Lori Marz, Vanadzor; Vandzor city centre	Recreational	22.04.2022	1343	40°48′07″N; 44°29′45″E
W-14	Water, River	**River Pambak**: Lori Marz, Vanadzor; site is downstream of Vanadzor city	Recreational	22.04.2022	1330	40°48′36″N; 44°30′27″E
W-15	Water, River	**River Pambak**: Lori Marz, Shenavan; site is upstream of Vanadzor city	Recreational, Agricultural	22.04.2022	1602	40°51′04″N; 44°14′06″E
W-16	Water, River	**River Herher**: Vayots Dzor Marz, Herher	Recreational	05.05.2022	1333	39°41′21″N; 45°31′18″E
W-17	Water, River	**River Hrazdan**: Kotayk Marz, Bjni	Recreational, Agricultural	16.05.2022	1440	40°26′13″N; 44°37′14″E
W-18	Water, River	**River Dalar**: Kotayk Marz, Aghveran;	Recreational	16.05.2022	1600	40°29′05″N; 44°35′47″E
W-19	Water, River	**River Aghstev**: Tavush Marz, Ijevan; site is downstream of Ijevan & Dilijan towns	Recreational	14.07.2022	632	40°55′32″N; 45°09′17″E
W-20	Water, River	**River Aghstev**: Tavush Marz, Dilijan; site is upstream of Dilijan and Ijevan towns	Recreational	14.07.2022	1280	40°44′01″N; 44°49′01″E
W-21	Water, Lake	**Lake Sevan**: Gegharkunik Marz, Shoghakat	Recreational	14.08.2022	1903	40°29′09″N; 45°16′47″E
W-22-1	Water, Lake	**Akhuryan reservoir**: Shirak Marz, Shirakavan	Agricultural	11.10.2022	1480	40°40′13″N; 43°45′26″E
W-22-2	Sediment, Lake					
W-23-1	Water, River	**River Aghstev**: Tavush Marz, Ijevan; site is downstream of Ijevan & Dilijan towns	Recreational	18.10.2022	632	40°55′32″N; 45°09′17″E
W-23-2	Sediment, River					
W-24	Water, River	**River Pambak**: Lori Marz, Vanadzor; site is downstream of Vanadzor city	Recreational	24.11.2022	1330	40°48′36″N; 44°30′27″E

a STW = Sewage treatment works.

b MASL = Metres above sea level.

### 2.2 Sample collection

Samples were collected from around 20–30 cm depth in each water body, approximately 2–3 m from the bank of the water body. Water samples were collected into clean 10 L plastic containers and sediment samples (approximately 100 g) were collected into clean plastic bags. Samples were transported in cool boxes to the Laboratory of Molecular Parasitology at the Scientific Center of Zoology and Hydroecology, National Academy of Sciences of the Republic of Armenia in Yerevan within 4 h of collection. Here they were stored refrigerated (4 °C) before processing commenced within 24–48 h.

### 2.3 Sample processing

#### 2.3.1 Water and sediment samples preparation

The water samples were shaken manually for around 5 s before filtering through polycarbonate membrane filters (diameter 47 mm; pore size 2.5 μm; REATRECK FILTER Ltd., Obninsk-3, Kaluga Region, Russian Federation). When the filter clogged, it was removed and refrigerated, and a new filter was used to continue the filtration. For each sample between two and 14 filters were required depending on the amount of particulate material in the sample. When the sample had been filtered, each membrane filter was split into two equal parts using a sterile scalpel.

The sediment samples were transferred to 15 mL centrifuge tubes and centrifuged for 3 min at 1500 rpm (430 *g*) and the supernatant removed. The sediment material was combined and stored refrigerated (+4 °C) in plastic tubes before transporting to the Norwegian University of Life Sciences for further analysis.

#### 2.3.2 Water sample analysis for *Cryptosporidium* oocysts by modified Ziehl-Neelsen staining

**Fig. 2:**
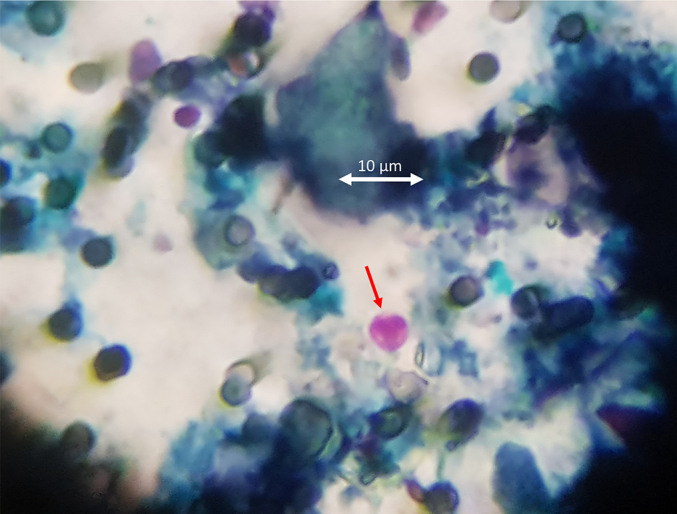
*Cryptosporidium* sp. oocyst (indicated by red arrow) on filter surface (stained by mZN) among other debris. (For interpretation of the references to colour in this figure legend, the reader is referred to the web version of this article.)

For 23 of the 24 water samples, one half of each filter for that sample was placed on a microscope slide and fixed in 96% ethanol, covering the whole filter for 10 min. The slide was flame-dried for 3–4 s, then stained using the standard mZN technique (e.g., Garcia et al., 1983; Casemore, 1991), with carbol fuschin stain, decolourisation with 3% hydrochloric acid, and Brilliant Green counterstain. The samples were then screened by light microscopy at x1600 (objective 100×, eyepiece 16×) using immersion oil and oocysts identified by their characteristic pink colouration and size (3–5 μm diameter) against a green background (see Fig. 2). The number of oocysts seen per sample was registered.

#### 2.3.3 Water sample analysis for *Giardia* cysts by Lugol staining

For one sample, one half of each filter used for concentrating the sample was stained with Lugol solution on a microscope slide, at the orientation that the filter collection side was upward, and Lugol solution added to cover the filter. The samples were then screened by light microscopy and *Giardia* cysts identified by morphological criteria. The number of cysts seen per sample was registered.

#### 2.3.4 Water sample analysis for *Cryptosporidium* and *Giardia* by immunofluorescent antibody test

For two of the 29 samples, one half of each filter used for that sample was placed on a microscope slide, at the orientation that the filter collection side was upward, and fixed by methanol solution added to cover the filter. The samples were then examined by immunofluorescent antibody test (IFAT), with the fixed sampled stained with commercially available (AquaGlo, Waterborne Inc., New Orleans, USA) monoclonal antibodies against *Cryptosporidium* oocyst walls and *Giardia* cyst walls, labelled with fluorescein isothiocyanate (FITC) by incubation at 37 °C for 30 min. Following incubation, the samples were stained with 4′,6-diamidino-2-phenylindole (DAPI), rinsed and then anti-fade mounting medium (1,4-diazabicyclo [2.2.2] octane; DABCO) added, before screening by fluorescence microscopy at x250 (and x400, if required) using appropriate filter blocks for visualising FITC and DAPI. *Cryptosporidium* oocysts and *Giardia* cysts were identified by characteristic fluorescent staining and morphological criteria. The number of parasites seen per sample was registered.

#### 2.3.5 Water sample analysis for *Cryptosporidium* and other parasites by qPCR

For 11 samples, chosen on the basis of mZN results (eight had been found to be positive for *Cryptosporidium* oocysts by mZN), one half of each filter was cut into small pieces using scissors or scalpel, and the pieces added into kit tubes for DNA extraction (BioFACT Genomic DNA Prep Kit, SmartScience Co. Ltd., Bangkok, Thailand), with between four and eight pieces of filter snippets per tube. DNA was extracted according to the manufacturer's protocol. The eluted DNA was frozen at −20 °C before transportation to the Norwegian University of Life Sciences for analysis by qPCR.

For each DNA eluate, qPCR was conducted for detection of DNA of *Cryptosporidium* spp., *Toxoplasma gondii, Echinococcus multilocularis*, and *Cyclospora cayetanensis* using the protocols described by Temesgen et al. (2022). This consists of one multiplex PCR using 2 μL of template (for *T. gondii, E. multilocularis*, and *C. cayetanensis;* Temesen et al., 2019) and a separate simplex PCR for *Cryptosporidium* using 5 μL template and the protocol of Elwin et al. (2022).

The qPCR was run in using a Stratagene AriaMxReal-Time PCR System (Agilent Technologies, Inc., Santa Clara, CA, US) with Agilent AriaSoftware v1.5., with all samples run in two technical replicates of duplicate samples, and using appropriate positive and negative controls in each run.

#### 2.3.6 Sediment sample analysis for *Cryptosporidium* oocysts and *Giardia* cysts by immunomagnetic separation followed by immunofluorescence antibody test

A mixed aliquot of approximately 5 g of each of the two sediment samples were resuspended in water and transferred to L10 tubes, together with the necessary buffers and beads for immunomagnetic separation (IMS) of *Cryptosporidium* oocysts and *Giardia* cysts using the Dynabeads Cryptosporidium/Giardia Combo Kit (Idexx, UK) according to the manufacturer's instructions. After mixing for 1 h, and following subsequent dissociation of the beads and potential parasites by vigorous shaking in 50 μL of 0.1 M hydrochloric acid, the suspension was added to a welled slide, neutralised by addition of 5 μL of 1 M sodium hydroxide and air-dried. The dried concentrate was fixed in methanol, then stained with AquaGlo as described above in Section 2.3.4, and examined as previously described for IFAT by fluorescence microscopy.

### 2.4 Statistics

Proportions were compared by contingency table analysis to investigate for any relevant differences between sample site and contamination.

## 3 Results

The results of the analyses are summarized in Table 2.

**Table 2:**
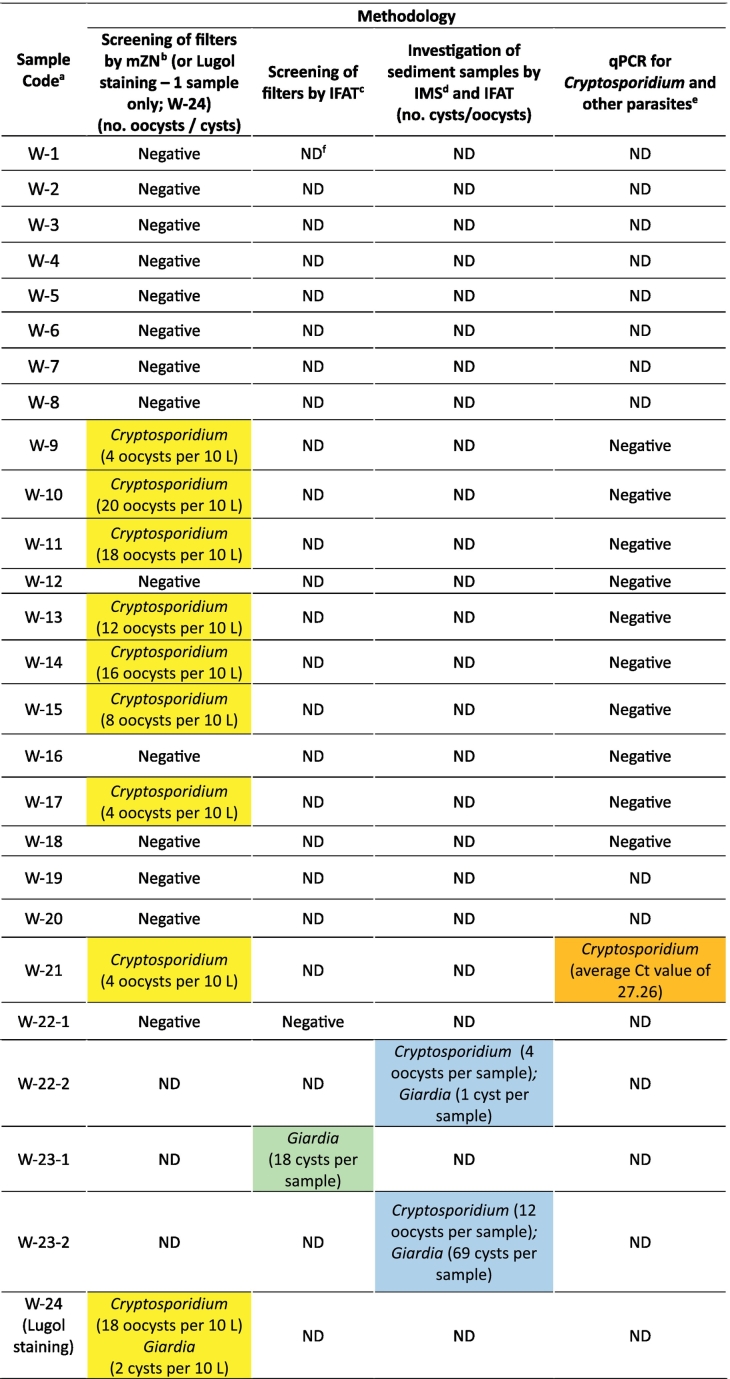
Overview of analysis results (according to location and analysis method).

^a^: Please refer to Table 1 for further information on each sample.

^b^: Modified Ziehl-Neelsen.

^c^: Immunofluorescent antibody test.

^d^: Immunomagnetic separation.

^e^: Other parasites tested for by multiplex qPCR: *Cyclospora cayetanensis, Echinococcus multilocularis, Toxoplasma gondii.*

^f^: ND = Not done.

**Shading**:

Yellow:water samples positive by mZN (*Cryptosporidium*) and/or Lugol staining (*Giardia*)

Green: water samples positive by IFAT (*Cryptosporidium* and/or *Giardia*)

Blue: sediment samples positive by IFAT (*Cryptosporidium* and/or *Giardia*)

Orange: Water samples positive by qPCR (*Cryptosporidium*)

Of the 23 water samples analysed by mZN, *Cryptosporidium* oocysts were detected in nine (39%). The number of oocysts in positive samples ranged from between four and 20 per 10 L. One of these samples was also analysed for *Giardia* cysts and found to be positive (two cysts per 10 L).

Of the two water samples examined by IFAT, one was negative for both *Cryptosporidium* oocysts and *Giardia* cysts and the other positive for *Giardia*, with 18 cysts per 10 L.

Of the 23 water samples analysed by mZN, 11 were also analysed by qPCR, eight of which had been found positive for *Cryptosporidium* by mZN analysis. DNA from *T. gondii, C. cayetanensis*, and *E. multilocularis* was not detected in any of these 11 samples, but *Cryptosporidium* DNA was detected in one sample that had also been found positive by mZN.

The two sediment samples analysed by IMS and IFAT were both positive for both parasites. In one sample (W-22-2), four *Cryptosporidium* oocysts and one *Giardia* cyst was detected, and in the other sample (W-23-2) 12 *Cryptosporidium* oocysts and 69 *Giardia* cysts were detected. This sediment sample had been collected from the same artificial lake as the one water sample (W-23-1) found to be positive for *Giardia* cysts by IFAT.

Statistical analyses revealed no associations regarding location, water type, or sampling period.

## 4 Discussion

The main finding of this study was that both *Cryptosporidium* oocysts and *Giardia* cysts occur widely in water bodies in Armenia, with some sediment samples indicating relatively high levels of contamination. A previous study from neighbouring Turkey (Samsun) has also reported high occurrence of *Cryptosporidium* and *Giardia* in water samples, with approximately 60% contaminated with each parasite (Karaman et al., 2017). The mixture of methods used for analysis in our study means that, in general, it is not possible to determine whether differences in results between samples reflect methodology used for analysis or differences in contamination rate between samples. It should be noted that samples taken from water bodies in agricultural areas had no significantly greater likelihood of being positive than samples from water bodies in recreational areas.

Although qPCR is considered to be significantly more sensitive than mZN microscopy for diagnostic detection of *Cryptosporidium* (e.g., Chalmers et al., 2011), this is not seen in our results. The reason for this could be that the oocysts were opened and dead and did not contain DNA for detection. Alternatively, it could be that the DNA extraction method was insufficiently vigorous to open the oocysts such that the primers could access the DNA; it is well established that oocyst shells are very robust and proteinase K treatment can be insufficient to open them to access the DNA. A step such as freeze-thawing, boiling, or bead-beating is necessary in the DNA extraction protocol (Robinson et al., 2020). This also means that the negative results for the other parasites tested for by qPCR (*T. gondii, E. multilocularis*, and *C. cayetanensis*) should also be treated with caution, as their transmission stages are also known to be highly robust.

In addition, no attempts were made to investigate the possibility of PCR inhibition, for example by use of an internal positive control.

Nevertheless, it should be noted that one water sample that was positive for *Cryptosporidium* by mZN was also found to be positive by qPCR. This sample, which did not have a particularly high oocyst count (by mZN), was collected from a popular recreational / bathing zone in Lake Sevan, with maybe over 100 visitors daily, particularly families with children, during the summer months when the sample was taken.

One result of particular interest was the relatively high levels of both *Cryptosporidium* oocysts and *Giardia* cysts detected in the two sediment samples, processed using IMS followed by IFAT for detection; neither of these samples were analysed by PCR. It is of interest that *Giardia* was also detected in a water sample obtained from one of the locations where the sediment samples were taken. These results that imply high levels of contamination in the water body sediment, potentially indicating repeated or continuous contamination of the water source, such that parasites have built up in the sediment. It is of interest to note that the site with the highest number of parasites (sampling site: W-23; River Aghstev) is also a popular recreational area, although not particularly used for bathing. Two relatively large tourist towns are located on the river; the sampling location was on the outskirts of Ijevan (ca. 18,000 inhabitants) and 30 km downstream from a larger town (ca. 30,000 inhabitants), Dilijan, also a tourist destination. For both towns, untreated sewage is discharged directly to the River Aghstev.

The lack of *Giardia*-positive water samples is likely to reflect that very few water samples were tested specifically for this parasite; only one filter was stained with Lugol and two by IFAT. Thus, of the five samples actually tested for *Giardia* (three water samples and two sediment samples), four were found to contain *Giardia* cysts.

This preliminary study has various limitations, including the mixture of methods used that precludes meaningful comparisons between sampling sites, and lack of optimisation of some methods (e.g., lack of use of an internal positive control in the PCR). In addition, the staining and microscopic analysis of membrane filters (either with mZN, Lugol, or IFAT) was difficult due to the amount of background matter on the membranes and the large area needing to be screened. Elution from the filters and concentration prior to fixing, staining, and screening, preferably with IFAT (in accordance with the ISO 15553 Standard method; ISO, 2006), may not only be more sensitive and produce more reliable results, but would also diminish the effort required in the analyses. A further limitation was the lack of investigation of the recovery efficiencies of the methods. However, this would have increased the microscopy burden and it would have been inappropriate to extrapolate recovery efficiency in one particular matrix to that of another. Nevertheless, by using various methods in this preliminary study not only was some initial data obtained, but the most useful approach to further studies within the Armenian context was understood.

Despite the various limitations, our preliminary findings indicate the importance of conducting a more detailed study of these parasites in the Armenian environment, and determining the most likely sources of contamination. In addition, collecting data on infection in people and animals with these parasites in catchment areas and following exposure to these water sources could also provide information of value that could be included in risk assessment. Such data could also be used for determining where measures could be implemented to reduce the risk of transmission of these parasites, especially as these very relevant data are currently lacking or very scanty in Armenia.

## Funding

This research was financially supported through Research Project #20TTCG-1F006 “Parasitic Protozoa in Surface Waters and Soils in Armenia: Investigation, Characterization, and Monitoring Approaches” funded by State Committee of Science, Republic of Armenia.

## Declaration of competing interest

Lucy J. Robertson reports a relationship with European Federation of Parasitologists that includes: board membership. Lucy J Robertson reports a relationship with Nord University that includes: travel reimbursement. Lucy J. Robertson reports a relationship with Norwegian Scientific Committee for Food Saftety that includes: travel reimbursement. LJR: Associate editor for FAWPAR. The other authors declare that they have no known competing financial interests or personal relationships that could have appeared to influence the work reported in this paper.
